# A prostacyclin analogue, iloprost, protects from bleomycin-induced pulmonary fibrosis in mice

**DOI:** 10.1186/1465-9921-11-34

**Published:** 2010-03-20

**Authors:** Yuanjue Zhu, Yong Liu, Weixun Zhou, Ruolan Xiang, Lei Jiang, Kewu Huang, Yu Xiao, Zijian Guo, Jinming Gao

**Affiliations:** 1 Department of Respiratory Diseases, Peking Union Medical College Hospital, Chinese Academy of Medical Sciences & Peking Union Medical College, Beijing 100730, China; 2 Department of Pathology, Peking Union Medical College Hospital, Chinese Academy of Medical Sciences & Peking Union Medical College, Beijing 100730, China; 3 Department of Physiology and Pathophysiology, Peking University Health Sciences Center, Beijing 100191, China; 4 Department of Respiratory Medicine, Chaoyang Hospital, Capital Medical University, Beijing 100020, China

## Abstract

**Background:**

Metabolites of arachidonic acid such as prostacyclin (PGI_2_) have been shown to participate in the pathogenesis of pulmonary fibrosis by inhibiting the expression of pro-inflammatory and pro-fibrotic mediators. In this investigation, we examined whether iloprost, a stable PGI_2 _analogue, could prevent bleomycin-induced pulmonary inflammation and fibrosis in a mouse model.

**Methods:**

Mice received a single intratracheal injection of bleomycin with or without intraperitoneal iloprost. Pulmonary inflammation and fibrosis were analysed by histological evaluation, cellular composition of bronchoalveolar lavage (BAL) fluid, and hydroxyproline content. Lung mechanics were measured. We also analysed the expression of inflammatory mediators in BAL fluid and lung tissue.

**Results:**

Administration of iloprost significantly improved survival rate and reduced weight loss in the mice induced by bleomycin. The severe inflammatory response and fibrotic changes were significantly attenuated in the mice treated with iloprost as shown by reduction in infiltration of inflammatory cells into the airways and pulmonary parenchyma, diminution in interstitial collagen deposition, and lung hydroxyproline content. Iloprost significantly improved lung static compliance and tissue elastance. It increased the expression of IFNγ and CXCL10 in lung tissue measured by RT-PCR and their levels in BAL fluid as measured by ELISA. Levels of TNFα, IL-6 and TGFβ1 were lowered by iloprost.

**Conclusions:**

Iloprost prevents bleomycin-induced pulmonary fibrosis, possibly by upregulating antifibrotic mediators (IFNγ and CXCL10) and downregulating pro-inflammatory and pro-fibrotic cytokines (TNFα, IL-6, and TGFβ1). Prostacyclin may represent a novel pharmacological agent for treating pulmonary fibrotic diseases.

## Introduction

Idiopathic pulmonary fibrosis (IPF) is a progressively fatal disorder characterized by inflammatory alveolitis and scarring in the pulmonary interstitium with loss of lung function; it is estimated that there is a 70% mortality within 5 years from initial diagnosis [1]. The current pharmacologic therapy for IPF is limited and there are no effective treatments [1]. The mechanisms underlying the pathogenesis of IPF include the accumulation of inflammatory cells in the lungs, and the generation of pro-inflammatory and pro-fibrotic mediators, resulting in alveolar epithelial cell injury and fibroblast hyperplasia, and eventually excessive deposition of extracellular collagen [2]. Searching for new agents to meet this unmet medical need is a priority.

There is accumulating evidence that bioactive metabolites of arachidoic acid (eicosanoids) may either contribute to or protect against lung fibrosis. Eicosanoids may regulate the fibroproliferative response directly through an action on lung resident cells and/or indirectly through modulating recruitment of inflammatory cells, release of mediators, and intracellular signaling pathways [3]. Leukotriene (LT) B_4_, a metabolite synthesized by 5-lipoxygenase (5-LO), was elevated in bronchoalveolar (BAL) fluid of patients with IPF [4] and deletion of 5-LO leading to a deficiency in sulphidopeptide-leukotriene production ameliorated bleomycin-induced fibrosis in mice [5]. In addition, antagonizing LTB_4 _receptor attenuated the lung fibrosis induced by bleomycin in mice by suppressing the production of inflammatory and fibrotic cytokines and by promoting the antifibrotic cytokine, IFNγ [6].

In contrast to LTB_4_, prostaglandin (PG) E_2 _generated by cyclooxygenase (COX)-2 pathway inhibited lung fibrosis by suppressing fibroblast proliferation and collagen synthesis [7]. The preventive and therapeutic effects of the administration of a PGE_2 _synthetic compound on lung fibrosis induced by bleomycin through anti-inflammatory mechanisms has been recently demonstrated [8].

PGI_2_, known as prostacyclin, is produced through the action of COX-2 and a membrane-anchored prostacyclin synthase and is secreted by alveolar type II cells in large quantities [9]. By specifically binding to a single G-protein coupled receptor (IP), PGI_2 _induces anti-inflammatory and anti-fibroproliferative activity through elevating intracellular cyclic adenosine monophosphate (cAMP) [9]. A decreased level of PGI_2 _was found in fibroblasts isolated from IPF patients [10]. PGI_2 _has been shown to inhibit migration, proliferation and collagen synthesis of fibroblasts in vitro[11,12]. Mice lacking COX-2-derived PGI_2 _or IP were more susceptible to developing severe pulmonary fibrosis in response to bleomycin than wild type mice in a PGE_2_-independent fashion [13]. Additionally, a synthetic prostacyclin agonist attenuated bleomycin-induced lung fibrosis in mice [14]. Besides, inhalation of a stable PGI_2 _analogue, iloprost, was shown to abrogate the allergic inflammation in animal model of asthma [15].

PGI_2 _may inhibit the development of lung fibrosis by controlling inflammation and fibrosis [9]. The aim of this study was to investigate the role of PGI_2 _by using intraperitoneal administration of iloprost in a mouse model of bleomycin-induced pulmonary fibrosis and the possible mechanism(s) by which PGI_2 _might mediate its effect.

## Materials and methods

### Mice and bleomycin injection

Mice with C57BL/6 background (6 to 8-week old; 20-25 g body weight) were maintained in a pathogen-free mouse facility. All experiments were performed according to international and institutional guidelines for animal care and were approved by the Animal Ethics Committee of Peking Union Medical College Hospital. Clean food and water were supplied with free access.

The adult male mice were anesthetized with pentobarbital intraperitoneally, followed by a single intratracheal injection of 3 mg/kg of bleomycin sulfate (Nippon Kayaku, Japan) in 50 μl of sterile phosphate-buffered saline (PBS). Control mice were injected with 50 μl of sterile PBS. In some experiments examining lung mechanics and cellular and biochemical characterization of BAL fluid, we used a smaller dose of bleomycin (2 mg/kg body weight) in order to avoid significant mortality.

Iloprost (200 μg/kg; Schering, Berlimed, Spain) dissolved in 500 μl of PBS was intraperitoneally administered 10-15 minutes prior to intratracheal injection of bleomycin. In some experiments, iloprost was given intraperitoneally 7 days after bleomycin treatment. The dosage of iloprost adopted in this investigation was optimized based on the series of preliminary studies, in which we found no effectiveness at the lower doses of iloprost of 100 and 150 μg/kg.

The mice were randomly allocated into four groups: 1. PBS (PBS) alone; 2. PBS+iloprost; 3. bleomycin; 4. bleomycin (Bleo)+iloprost.

### Histopathological evaluation of pulmonary fibrosis

On day 14 post-administration, animals were sacrificed by overdosage of pentobarbital and perfused via the left ventricle with 5 ml of cold saline. The lungs were carefully removed, inflated to 25 cmH_2_O with 10% formalin and fixed overnight, embedded in paraffin, and sectioned at 5 μM thickness. The sections were stained with Hematoxylin & Eosin for routine histology or with Masson trichrome for mature collagen.

Histopathological scoring of pulmonary fibrosis was performed as described by Ashcroft and co-workers [16]. The severity of fibrotic changes in each lung section was assessed as a mean score of severity. At least 10 high-power fields within each lung section were evaluated.

Alveolar septal thickening was quantified using digital imaging as previously described [17]. Briefly, at least five images of representative areas of each lung lobe stained with hematoxylin and eosin were randomly captured and analyzed for alveolar thickening, accumulation of leukocytes, and increased extracellular matrix and fibroblasts. With NanoZoomer Digital Pathology C9600 (Hamamatsu Photonics K.K., Japan), threshold was defined as the areas containing thickened septum of digital images which were automatically counted by the system. Then the threshold areas were divided by the total areas of the selected images and multiplied by 100 to generate a percentage of the thickened area in each mouse.

The pathological analysis was independently performed for each mouse in a blind manner by two experienced pathologists.

### Assessment of lung mechanics

On day 21 after treatment, mice were prepared as previously described for invasive analysis of lung mechanics using a computer-controlled small animal ventilator, the Flexivent system (Scireq, Montreal, PQ, Canada)[13,18,19]. Briefly, mice were mechanically ventilated at a rate of 150 breaths/min, tidal volume of 10 ml/kg, and a positive end-expiratory pressure of 3 cmH_2_O. We documented the tracheal pressure (Ptr), volume (V), and airflow. Pressure-volume curves were generated after delivering incremental air into lungs from functional residual to total lung capacity. Static compliance (Cst), reflecting elastic recoil of the lungs, was calculated by the Flexivent software using Salazar-Knowles equation. Tissue elastance (H) was measured by forced oscillation technique using Flexivent software.

### Bronchoalveolar lavage fluid

Bronchoalveolar lavage (BAL) fluid was conducted as previously described [20]. Briefly, mice were sacrificed 14 days later, and the trachea was cannulated by using 20-gauge catheter. BAL was performed three times with 0.8 ml of ice-cold PBS (PH 7.4) with 90% of recovery rate. The BAL fluid was spun, supernatant was collected and kept at -70°C until used. Recovered total cells were counted on a hemocytometer in the presence of 0.4% trypan blue (Sigma, MO). For differential cell counting, cells were spun onto glass slides, air-dried, fixed, and routinely stained. The number of macrophages, neutrophils and lymphocytes in 200 cells was counted based on morphology.

### Hydroxyproline assay

Total lung collagen was determined by analysis of hydroxyproline as previously described [21]. Briefly, lungs were harvested 14 days after treatment and homogenized in PBS (PH 7.4), digested with 12N HCl at 120°C overnight. Citrate/acetate buffer (PH 6.0) and chloramine-T solution were added at room temperature for 20 minutes and the samples were incubated with Ehrlich's solution for 15 min at 65°C. Samples were cooled to room temperature and read at 550 nm. Hydroxyproline standards (Sigma, MO) at concentrations between 0 to 100 μg/ml were used to construct a standard curve.

### RT-PCR analysis for mRNA expression of cytokines andchemokines

Total RNA was extracted from the lung using TRIzol reagent (Invitrogen, CA) according to manufacturer's instructions, and treated with RNase-free DNase. RNA was reverse-transcribed into cDNA using M-MuLV reverse transcriptase (Invitrogen). Then 1 μl of cDNA was subjected to PCR in a 25 μl final reaction volume for analysing the expression of CXCL10/IP-10, IL-6, TGFβ 1, and TNFα. β-actin was analysed as an internal control. The amplification conditions were as follows: initial step at 95°C for 10 min, followed by 35 cycles of 95°C for 1 min, 55°C for 1 min and 72°C for 1 min. The primers and products of RT-PCR are presented in Table 1.

**Table 1:** RT-PCR primers and products

Genes	S/AS	Primer sequence (5' to 3')	Products (bp)
**CXCL10**	S AS	GTCATTTTCTGCCTCATCC GAGCCCTTTTAGACCTTTT	273
**IL-6**	S AS	TGGGACTGATGCTGGTGA CTGGCTTTGTCTTTCTTGTTATC	376
**TGFβ1**	S AS	CCCTGTATTCCGTCTCCTT GCGGTGCTCGCTTTGTA	363
**TNFα**	S AS	GGCGGTGCCTATGTCTC GCAGCCTTGTCCCTTGA	383
**β-actin**	S AS	CTTCCTTAATGTCACGCACGATTTC GTGGGGCGGCCCAGGCACCA	541

S, sense; AS, antisense

### Analysis of cytokines, chemokines, and eicosanoids in BALF

The concentrations of IFNγ, IL-6, TGFβ1, and CXCL10/IP-10 in BAL fluid were determined by ELISA. The ELISA kits for IFNγ and CXCL10/IP-10 were purchased from R&D systems, the kits for IL-6 and TGFβ1 were products of Amersham Bioscience. The detection limits of IFNγ, IL-6, TGFβ1, and CXCL10/IP-10 were 4, 4, 60, and 2.2 pg/ml, respectively.

The levels of LTB_4 _and PGE_2 _were quantified using enzyme immunoassay (EIA) kits (Cayman chemical, MI). The detection limits for LTB_4 _and PGE_2 _were 15.3 pg/ml and 15.5 pg/ml, respectively.

### Statistics

Data are expressed as means ± SEM. Comparisons were carried out using ANOVA followed by unpaired Student's *t *test. Survival curves (Kaplan-Meier plots) were compared using a log rank test (Graph Pad Software Inc., San Diego, CA). A value of *P *less than 0.05 was considered significant.

## Results

### Effect of iloprost on survival rate and body weight loss

**Figure 1:**
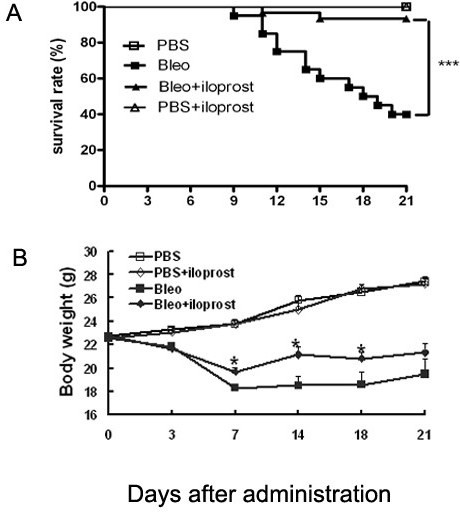
**Effect of iloprost on survival rate and body weight loss**. Mice were intratracheally injected with 3 mg/kg of bleomycin (Bleo) (no iloprost) or Bleo+iloprost (200 μg/kg). In the mice treated with Bleo (no iloprost), the mortality was as high as 60% by day 21; by contrast, the mortality was only 10% in the mice treated with Bleo+iloprost (A). Body weight loss was significantly attenuated in the mice treated with Bleo+iloprost in comparison with those treated with Bleo (no iloprost) (B). Results are expressed as mean ± SEM, n = 20 mice per group, *, p < 0.05, ***, p < 0.0001.

To demonstrate the protective effect of PGI_2 _on bleomycin-induced pulmonary injury, the mice were intraperitoneally administered with or without iloprost prior to injection of bleomycin at a dose of 3 mg/kg. The mice treated with bleomycin (but not receiving iloprost) began to die at day 9. Cumulative mortality was 60% at day 21; by contrast, mortality of mice treated with bleomycin+iloprost was significantly lower (10% at day 21, P < 0.0001) (Figure 1A). A protective effect of iloprost was also observed on weight loss. The mice treated with bleomycin (but not receiving iloprost) lost more weight than the mice treated with bleomycin+iloprost (Figure 1B).

### Effect of iloprost on bleomycin-induced pulmonary inflammation and fibrosis

**Figure 2:**
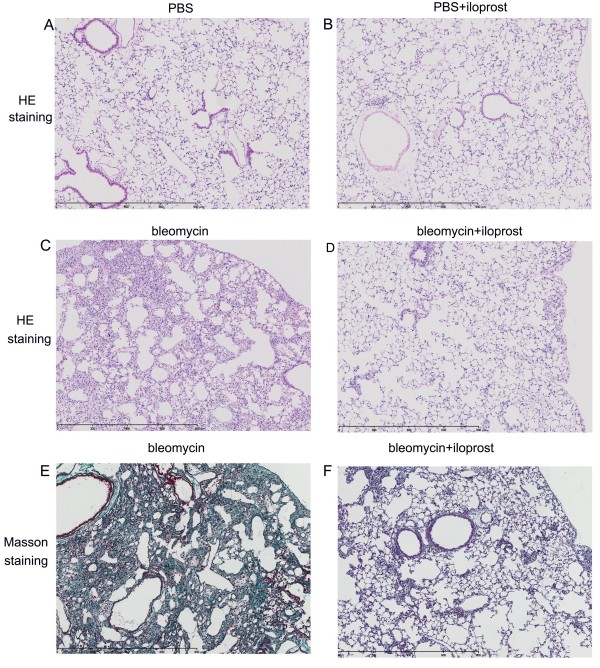
**Effect of iloprost on bleomycin-induced pulmonary inflammation and fibrosis**. Histological analysis of lungs in the mice treated with bleomycin and those treated with bleomycin+iloprost. Mice were killed at day 14, lungs were removed, inflated with 1 ml of 10% formalin. In the mice treated with PBS (no iloprost) or PBS+iloprost, there was normal alveolar structure (A and B). In the mice treated with bleomycin (no iloprost), there was more accumulation of leukocytes, distortion of alveolar architecture, and deposition of collagen (C and E), compared with the mice treated with bleomycin+iloprost (D and F). Panels A-D, H&E staining; Panel E-F, Masson's trichrome staining.

The effect of iloprost against bleomycin-induced fibrosis and inflammation was examined. Animals were sacrificed at day 14 after treatment and the lung sections were analyzed for the severity of inflammation and fibrosis. As shown in Figure 2, normal alveolar structure was seen in PBS-treated mice and PBS+iloprost-treated mice (A and B). Figure 2 shows representative lung histology at day 14 post-bleomycin installation. Mice treated with bleomycin (no iloprost) had more severe and extensive inflammation and fibrosis and more obvious alveolar wall thickening, distorted pulmonary architecture, massive infiltration of leukocytes and excessive deposition of mature collagen in interstitium (C and E), compared with the mice administered with bleomycin+iloprost (D and F).

**Figure 3:**
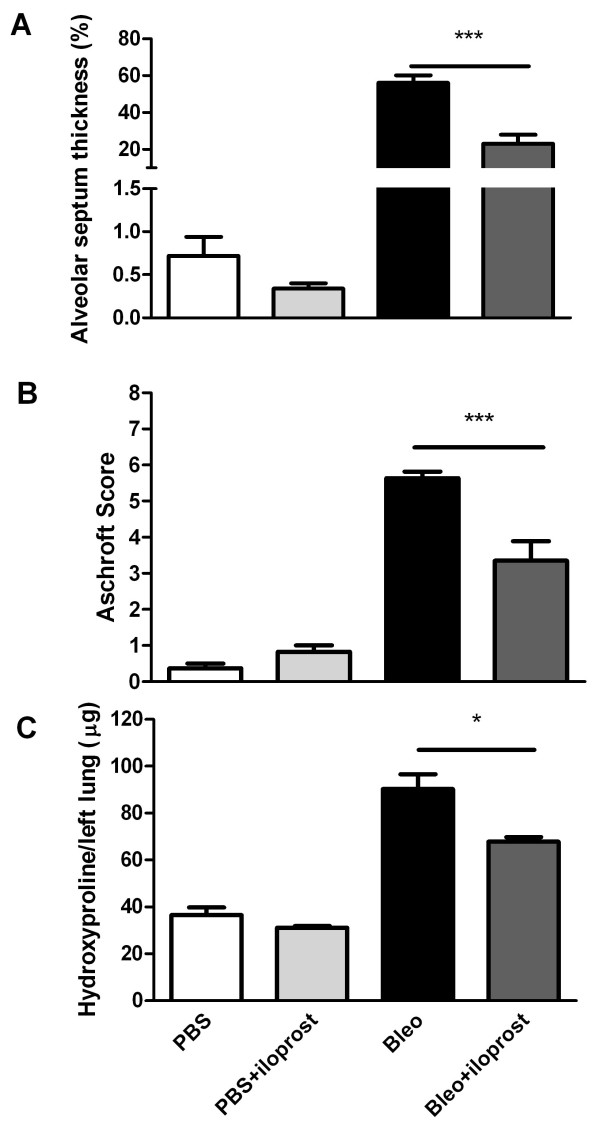
**Effect of iloprost on thickened areas of alveolar septum, histopathological scorings and hydroxyproline content in lung tissue**. A. Using digital imaging, the thickened areas of alveolar septum in the mice treated with Bleo (no iloprost) was significantly increased compared to those with Bleo+iloprost at day 14. Results are expressed as mean ± SEM, n = 6-8 mice per group, ***p < 0.001. B, semi-quantitative assessment was performed on day 14 using Aschroft scoring method, a significantly higher score was observed in the mice treated with Bleo (no iloprost) than those treated with Bleo+iloprost. Results are expressed as mean ± SEM, n = 5-8 mice per group, *** p < 0.001. C, the hydroxyproline content in lung tissue was significantly higher in the mice treated with Bleo (no iloprost) than those treated with Bleo+iloprost. Results are expressed as mean ± SEM, n = 5-7 mice per group, * p < 0.05.

We measured the thickened areas of alveolar septum relative to the total area of lung by digital imaging in at least five photographs of the lower lobes of lungs of the mice at day 14 post-treatment. PBS- or iloprost+PBS-treated mice had normal alveolar septa, and all scored less than 1%. The area of lungs with thickened alveolar septa treated with bleomycin (no iloprost) was 2.5-fold greater than in the mice treated with iloprost+bleomycin (56.1 ± 4.1% vs 23.0 ± 4.9%, P = 0.0004) (Figure 3A). There were significantly higher histopathologic scores in the mice treated with bleomycin (no iloprost) than in the mice treated with iloprost+bleomycin (5.64 ± 0.18 vs 3.35 ± 0.54, P < 0.0001) (Figure 3B).

To quantitatively assess the difference in extent of pulmonary fibrosis in the bleomycin-treated mice with or without iloprost, we assayed the hydroxyproline content unique to mature collagen in the lung tissue. The amount of hydroxyproline was significantly greater in bleomycin-treated mice than in iloprost+bleomycin treated-mice (90.29 ± 6.25 vs 67.84 ± 1.88 μg/left lung, P = 0.02) (Figure 3C).

### Effect of iloprost on infiltration of the inflammatory cells in airways

**Figure 4:**
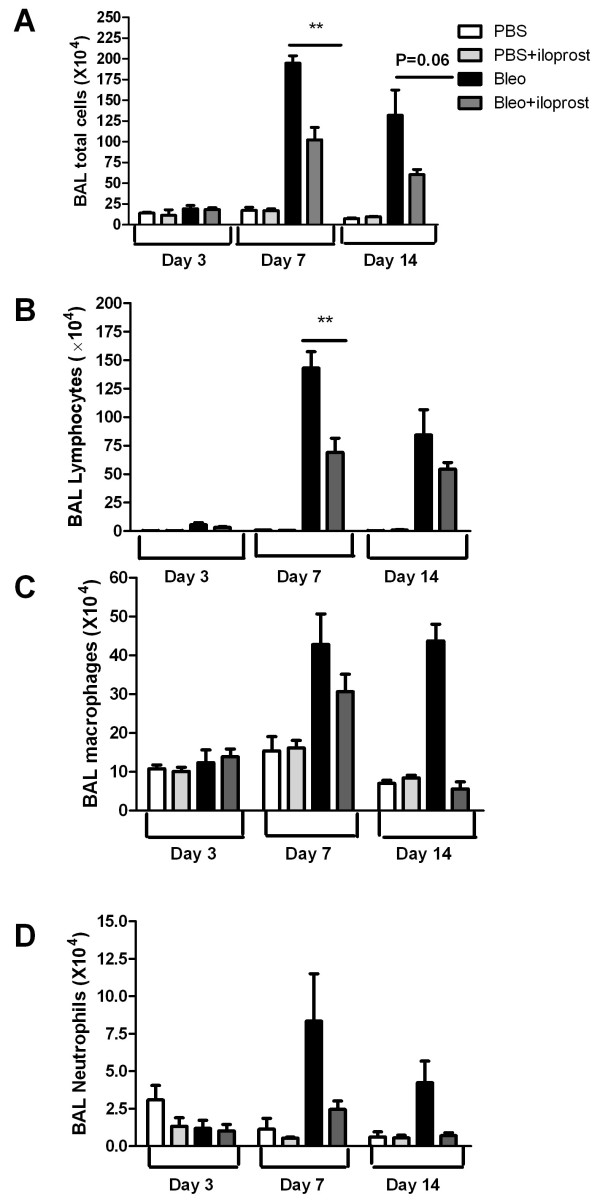
**Effect of iloprost on infiltration of inflammatory cells into the airways after bleomycin (Bleo) injection**. The mice were injected with 2 mg/kg of Bleo (no iloprost) or bleo+iloprost, BAL fluid was collected at days 3, 7, and 14 later. The number of inflammatory cells and lymphocytes accumulated in airways was significantly higher in the mice treated with Bleo (no iloprost) than those treated with Bleo+iloprost (A and B), and there was no significant difference in the number of macrophages and neutrophils in BAL fluid between the mice treated with Bleo (no iloprost) and those treated with Bleo+iloprost (C and D). Results are expressed as mean ± SEM, n = 5-8 mice each group, ** P < 0.01.

To determine whether iloprost affects bleomycin-induced infiltration of inflammatory cells into the airways, we estimated the cell populations in BAL fluid differentially 3, 7, and 14 days after bleomycin treatment. At day 7, the number of total inflammatory cells in BAL fluid was significantly less in the mice administrated with iloprost+bleomycin than those treated with bleomycin (no iloprost) (102.4 ± 14.9 × 10^4 ^vs 194.8 ± 9.0 × 10^4^, P < 0.01) (Figure 4A). At day 14, the total cells were marginally fewer in the mice treated with iloprost+bleomycin than those treated with bleomycin (no iloprost) (60.5 ± 6.2 × 10^4 ^vs 132.0 ± 30.7 × 10^4^, P = 0.06).

As represented in Figure 4A, the peak cellular response occurred at day 7 after bleomycin injection. The predominant cell type was the lymphocyte and the number of lymphocytes, not neutrophils and macrophages, was significantly greater in the mice treated with bleomycin (no iloprost) than in those treated iloprost+bleomycin (143.2 ± 14.3 × 10^4 ^vs 69.0 ± 12.5 × 10^4^; P < 0.01) (Figure 4B-C).

### Effect of iloprost on alteration of lung mechanics

**Figure 5:**
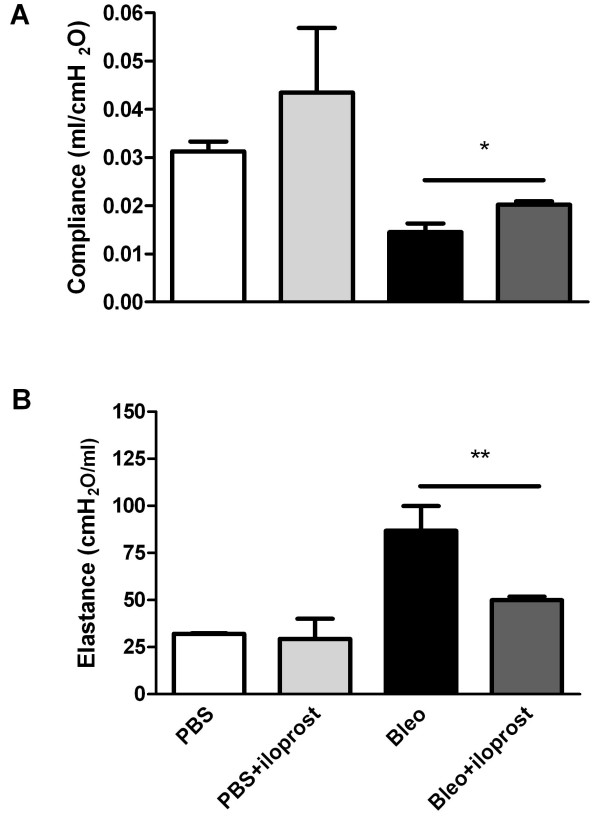
**Effect of iloprost on alteration of lung mechanics induced by bleomycin (Bleo)**. Measurement of lung function was performed at 21 days after injecting 2 mg/kg of bleomycin. Decrease in static compliance (Cst) (A) and increase in tissue elastance (H) (B) were significantly attenuated in the mice treated with Bleo+iloprost. Results are expressed as mean ± SEM, n = 10-12 mice per group, * P < 0.05, ** P < 0.01.

We measured static compliance and tissue elastance in accordance with the previous studies showing a decrease in static compliance (Cst) and increase in tissue elastance (H) in mice following bleomycin injury [13]. We found significant alterations of lung mechanics in mice treated with bleomycin (no iloprost), compared to control mice treated with PBS (no iloprost). However, decrease in Cst and increase in H were significantly attenuated in the mice treated with bleomycin+iloprost (for Cst: 0.014 ± 0.002 ml/cmH_2_O vs 0.020 ± 0.001 ml/cmH_2_O, P = 0.01; for H: 86.84 ± 13.11 ml/cmH_2_O vs 49.96 ± 1.83 ml/cmH_2_O, P < 0.01) (Figure 5A and 5B).

### Effect of iloprost on cytokines, chemokines and arachidonicacid products

**Figure 6:**
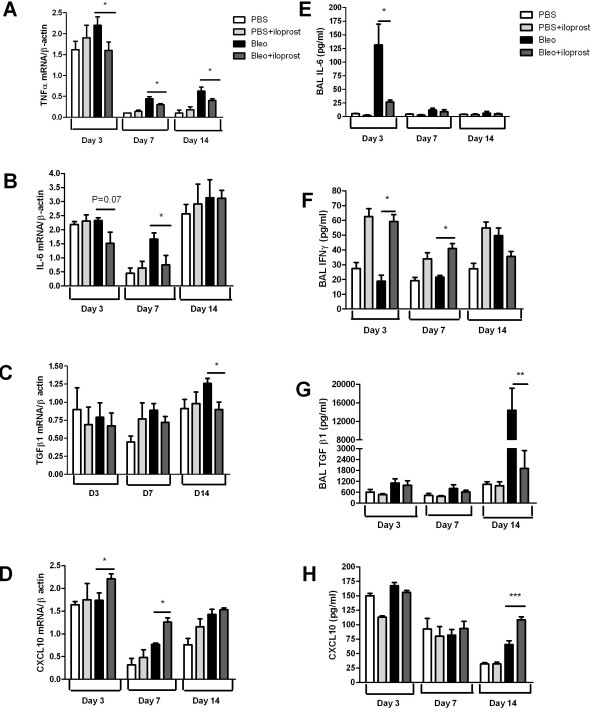
**Effect of iloprost on cytokines and chemokines at mRNA and protein levels**. BAL fluid and lung tissue were harvested at day 14 after injection of 2 mg/kg of bleomycin (Bleo) (no iloprost) or bleo+iloprost. mRNA expression of cytokines and CXCL10 was analyzed by semi-quantitative RT-PCR. The concentration of cytokines and CXCL 10 in BAL fluid was assayed by ELISA. Panels A-D show that mRNA expression of TNFα, IL-6, TGFβ_1_, and CXCL10 in lung tissue, n = 5-8 mice per group, * P < 0.05. Panels E-H show that the concentration of IL-6, IFNγ, TGFβ_1_, and CXCL10 in BAL fluid, n = 5-9 mice per group, * P < 0.05, ** P < 0.01, ***P = 0.001.

The level of TNFα mRNA was significantly lower in the mice treated with bleomycin+iloprost than the mice treated with bleomycin (no iloprost) (Figure 6A). IL-6 mRNA expression was decreased at day 3 and significantly lowered by day 7 in the mice treated with iloprost+bleomycin (Figure 6B), while TGFβ1 mRNA was significantly inhibited at day 14 (Figure 6C). CXCL10/IP-10 mRNA was significantly increased in lungs of the iloprost+bleomycin-treated mice by day 7 (P = 0.03 for day 3; P = 0.02 for day 7), and remained elevated at day 14 (Figure 6D).

ELISA assays determined that the level of IL-6 protein in BAL fluid was markedly elevated 3 days after bleomycin administration (no iloprost), but was significantly lower in mice treated with bleomycin+iloprost (131.5 ± 38.2 pg/ml vs 26.5 ± 4.0 pg/ml, P = 0.02) (Figure 6E). The concentration of IFNγ in BAL fluid was significantly higher at day 3 and remained elevated at day 7 in the mice treated with bleomycin+iloprost, compared with the mice treated with bleomycin (no iloprost) (at day 3: 59.3 pg/ml ± 10.5 pg/ml vs 18.9 ± 9.8 pg/ml, P = 0.02; at day 7: 41.0 ± 8.8 pg/ml vs 21.7 ± 2.5 pg/ml, P = 0.06) (Figure 6F). The concentration of TGFβ1 was significantly higher in BAL fluid recovered from the mice treated with bleomycin (no iloprost) than from the mice treated with iloprost+bleomycin at day14 (14350 ± 4798 pg/ml vs 1906 ± 990 pg/ml, P < 0.01) (Figure 6G). The concentration of IFNγ-inducible CXCL10 in BAL fluid was markedly higher in the mice treated with bleomycin+iloprost than those treated with bleomycin (no iloprost) at day 14 (108.4 ± 5.5 vs 65.9 ± 6.4 pg/ml, P = 0.001) (Figure 6H).

**Figure 7:**
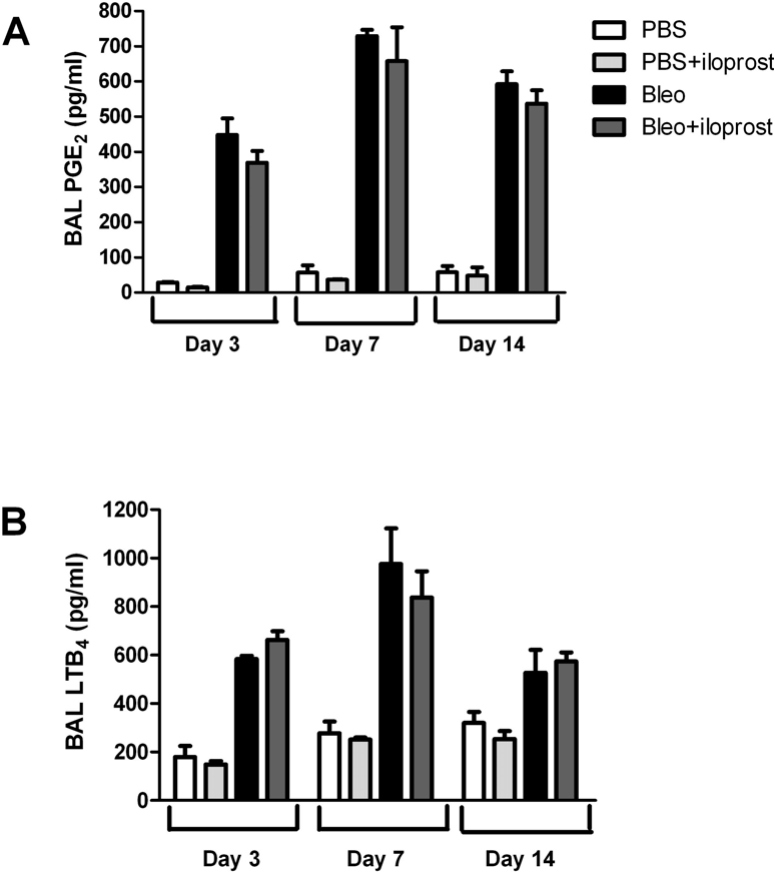
**Effect of iloprost on the production of PGE_2 _and LTB_4_**. BAL fluid was collected at day 14 after 2 mg/kg of bleomycin (Bleo) alone or Bleo+iloprost. The concentration of PGE_2 _(A) and LTB_4 _(B) in BAL fluid was measured by EIA, n = 5-8 mice per group.

The levels of PGE_2 _and LTB_4 _in BAL fluid were significantly higher in the mice treated with bleomycin compared with those treated with PBS. However, we found that LTB_4 _and PGE_2 _levels did not differ between the mice treated with bleomycin (no iloprost) and those treated with bleomycin+iloprost (Figure 7A and 7B).

### Effect of delayed application of iloprost on bleomycin-induced injury

When iloprost was given at day 7 post-bleomycin insult, we found that iloprost did not prolong the survival rate, did not improve the body weight loss, did not alleviate infiltration of the inflammatory cells, and did not decrease interstitial collagen accumulation in mice by day 21 post-bleomycin injection.

## Discussion

To our knowledge, this is the first report of an intraperitoneal application of iloprost, a PGI_2 _analogue, that prevented the pulmonary inflammation and fibrosis induced by bleomycin in mice. A single dose of iloprost prior to bleomycin injection significantly resulted in: (i) reduced mortality and body weight loss; (ii) attenuated infiltration of inflammatory cells into the lung and reduced collagen deposition in pulmonary interstitium; (iii) alleviation of the reduced static compliance and elevated tissue elastance; and (iv) a decreased production of proinflammatory and fibrotic cytokines such as TNFα, IL-6 and TGF-β1, and an increased release of antifibrotic mediators including IFNγ and chemokine CXCL10/IP-10.

Intratracheal instillation of bleomycin induces an acute pneumonitis with inflammatory cells aggregating in the pulmonary interstitium followed by aberrant fibroproliferation and collagen production in mice [22]. Our data showed that the influx of lymphocytes, other than macrophages and neutrophils, into lungs was considerably inhibited by iloprost at day 7 following bleomycin injection. These results suggest that iloprost might exert a direct inhibition of lymphocytic infiltration. Arras and colleagues have demonstrated that B lymphocytes are critical for lung fibrosis through the regulation of PGE_2 _in mice [23]. Another study performed in ovalbumin-sensitized mice indicated that iloprost had a direct inhibitory effect on lung dendritic cells, but with no effect on T helper 2 lymphocytes [15]. However, we were not able to determine in this current study which subtype of the inflammatory cells, such as natural killer cells and B cells, could be specifically suppressed by iloprost after bleomycin stimulation.

TNFα is considered to be one of the most potent proinflammatory cytokines promoting infiltration of inflammatory cells and proliferation of fibroblasts [24,25]. We showed in this study that induction of TNFα mRNA was markedly reduced in the mice treated with bleomycin and iloprost over the time-course of bleomycin-induced lung injury. A previous study reported that PGI_2 _analogues including iloprost decreased TNFα production by bone marrow-derived dendritic cells [26], and therefore the reduced mRNA expression of TNFα may result from this inhibitory effect of PGI_2_. IL-6 may modulate pulmonary inflammation as supported by the observation that an increased IL-6 level in BAL fluid was associated with lung fibrosis in human and animal models [27]. In addition, bleomycin-induced lung fibrosis was significantly attenuated in mice lacking the IL-6 gene [28]. In support of these results, our data showed that the IL-6 level in BAL fluid was elevated in mice 3 days after bleomycin injection; however, such increase was markedly abrogated in iloprost-treated mice. Our data implies that iloprost effectively inhibited the release of IL-6 from the infiltrated inflammatory cells at the initial stage of bleomycin-induced lung injury.

TGFβ_1_, a fibrogenic cytokine, is expressed in a variety of cells including fibroblasts, macrophages, and epithelial and endothelial cells [29,30]. Evidence from human studies and animal models indicates that TGFβ_1_, up-regulated in the process of fibrosis, plays a pivotal role in mediating the progression of the fibrotic diseases by stimulating fibroblasts to synthesize extracellular matrix proteins [31,32]. Sime and colleagues demonstrated that rats overexpressing active TGFβ_1 _gene developed marked lung fibrosis at day 14 [33]. Consistent with these observations, we observed that TGFβ_1 _mRNA and protein was significantly inhibited in the mice treated by iloprost+bleomycin at day 14. As represented in Figure 6, the increase in IL-6 in BAL fluid at early stage and the elevation of TGFβ_1 _in BAL fluid at the late stage of bleomycin-induced pulmonary injury may support previous reports indicating that IL-6 may regulate TGFβ_1 _signaling [34]. Collectively, these studies indicate that the involvement of PGI_2 _in preventing lung fibrosis may be due to its direct inhibitory effect on cellular immune response, leading to a reduction in fibrotic mediators.

There is substantial evidence supporting a key role of inhibitory modulators such as the Th1 cytokine, IFNγ, against fibroblast activation, [35]. A relative deficiency in IFNγ mRNA expression was associated with progressive lung fibrosis in IPF patients [36]. Exogenous administration of IFNγ has been shown to be critical for limiting lung fibrosis in CXCR3 knockout mice lacking endogenous IFNγ [37]. An in vitro study has suggested that IFNγ exerts the inhibitory effect on TGFβ_1 _signaling pathways [38]. In this study, we reported that IFNγ levels were markedly higher in the mice treated with iloprost and bleomycin than those treated with bleomycin without iloprost. Interestingly, we first observed that iloprost significantly induced production of IFNγ in PBS treated-mice by day 14 in this current study (Figure 6F), indicating that PGI_2 _is capable of upregulating anti-fibrotic mediators such as IFNγ. Additionally, an in vivo study examining the changes of biomarkers in IPF patients indicated that IFNγ may modulate fibrosis by down-modulating several pathways relevant to fibrosis, angiogenesis, proliferation, and immunoregulation [39]. The exact regulatory mechanism of PGI_2 _on IFNγ needs further investigation.

CXCL10/IP-10, which is regulated by the antifibrotic factor IFNγ, has been shown to attenuate bleomycin-induced pulmonary fibrosis in mice via inhibition of fibroblast recruitment or of angiogenesis [40]. CXCL10-deficient mice displayed increased fibroblast accumulation in the lung after bleomycin exposure. Conversely, transgenic mice overexpressing CXCL10 were less likely to die after bleomycin exposure, associated with a reduction in fibroblast accumulation in the lung [41,42]. Our data demonstrated that there was an increase in CXCL10/IP-10 mRNA level by day 7 and at the protein level at day 14 in the mice treated with iloprost and bleomycin as compared to those treated with bleomycin (no iloprost). We cautiously propose that induction of CXCL10/IP-10 could be secondary to the effect of IFNγ which was up-regulated by iloprost in our investigation; however, we cannot rule out other pathways modulating CXCL10/IP-10 in response to bleomycin.

An alternative explanation for both the reduced inflammatory and fibrotic response to bleomycin by iloprost in mice could be eicosanoid imbalance favoring the overproduction of antifibrotic prostaglandins (PGE_2_) and underproduction of fibrotic leukotrienes (LTB_4_). PGE_2 _is generally recognized as a potent anti-fibrotic agent, and is a major eicosanoid product of alveolar epithelial cells, macrophages, and fibroblasts [43,44]. Deficiency in PGE_2 _has been linked to severity of lung injury and fibrosis [23,45]. The production of PGE_2 _significantly rose in BAL fluid after intratracheal instillation of bleomycin; however, the increase seen in the mice treated with iloprost and bleomycin was similar to those treated with bleomycin (no iloprost) in this current model. Lovgren and coworkers using mice lacking COX-2 and IP demonstrated that PGE_2 _was not involved in the protection against bleomycin-induced lung fibrosis provided by prostacyclin [13]. Thus, a PGI_2_-mediated mechanism of preventing lung fibrosis induced by bleomycin is likely to be unrelated to PGE_2_. Leukotriene B_4 _functions as a proinflammatory and profibrotic mediator by binding to its specific receptor [6]. Iloprost did not modulate the increase in LTB_4 _levels in BAL fluid in response to bleomycin, suggesting that iloprost may not affect the lipoxygenase pathway and that iloprost does not limit bleomycin-induced lung pathology by inhibition of LTB_4_.

In this study, iloprost given at day 7 post-bleomycin, the time point at which pneumonitis and fibrosis are established, failed to decrease mortality and weight loss, to attenuate inflammation and to reverse lung fibrosis in bleomycin-treated mice by day 21. These data may indicate that iloprost can be preventive, but possibly not therapeutic, for lung fibrotic diseases. It must be emphasized that iloprost was given at one single dose by intraperitoneal route in our study, and therefore additional studies are necessary to test for a reversal effect of iloprost in a time- and dose-dependent fashion, e.g. when given 2-3 days after bleomycin injection and with repeated doses. In addition, whether long-term treatment with iloprost administered via the inhaled route would be beneficial for patients with lung fibrotic diseases should be further investigated.

## Conclusions

In conclusion, these observations provide evidence for a beneficial role of PGI_2 _in dampening pulmonary inflammation and fibrosis, possibly through inhibiting recruitment of inflammatory cells (predominantly lymphocytes) and decreasing production of TNFα, IL-6 and TGFβ1, while promoting the generation of IFNγ and IFNγ-targeted CXCL10/IP-10, which are anti-fibroproliferative.

## Conflict of interest statement

The authors declare that they have no competing interests.

## Authors' contributions

YZ co-supervised and involved in the whole experiment; YL performed the whole experiment; WZ and YX carried out the pathological analysis; RX, LJ and ZG helped and did some experiments; KH performed the lung function assay; JG designed and co-supervised the experiments, and drafted the manuscript. All authors have read and approve the final manuscript.
